# Effect of *Achillea fragrantissima* Extract on Excision Wound Biofilms of MRSA and *Pseudomonas aeruginosa* in Diabetic Mice

**DOI:** 10.3390/ijms24119774

**Published:** 2023-06-05

**Authors:** Yasir Almuhanna, Mohammed Hussein Alqasmi, Hamood AlSudais, Mohammed Alrouji, Fahd A. Kuriri, Mohammed Alissa, Meshari A. Alsuwat, Mohammed Asad, Babu Joseph

**Affiliations:** 1 Department of Clinical Laboratory Sciences, College of Applied Medical Sciences, Shaqra University, Shaqra 11961, Saudi Arabia; 2 Chair of Medical and Molecular Genetics Research, Department of Clinical Laboratory Sciences, College of Applied Medical Sciences, King Saud University, Riyadh 12372, Saudi Arabia; 3 Department of Medical Laboratory Sciences, College of Applied Medical Sciences, Prince Sattam Bin Abdulaziz University, Al-Kharj 11942, Saudi Arabia; 4 Clinical Laboratory Sciences Department, College of Applied Medical Sciences, Taif University, Al-Taif 21974, Saudi Arabia

**Keywords:** HaCaT cell lines, diabetic, cytotoxicity, skin irritation, LC-MS

## Abstract

*Achillea fragrantissima*, a desert plant commonly known as yarrow, is traditionally used as an antimicrobial agent in folklore medicine in Saudi Arabia. The current study was undertaken to determine its antibiofilm activity against methicillin-resistant *Staphylococcus aureus* (MRSA) and multi-drug-resistant *Pseudomonas aeruginosa* (MDR-*P. aeruginosa*) using in vitro and in vivo studies. A biofilm model induced through an excision wound in diabetic mice was used to evaluate its effect in vivo. The skin irritation and cytotoxic effects of the extract were determined using mice and HaCaT cell lines, respectively. The *Achillea fragrantissima* methanolic extract was analyzed with LC-MS to detect different phytoconstituents, which revealed the presence of 47 different phytoconstituents. The extract inhibited the growth of both tested pathogens in vitro. It also increased the healing of biofilm-formed excision wounds, demonstrating its antibiofilm, antimicrobial, and wound-healing action in vivo. The effect of the extract was concentration-dependent, and its activity was stronger against MRSA than MDR-*P. aeruginosa*. The extract formulation was devoid of a skin irritation effect in vivo and cytotoxic effect on HaCaT cell lines in vitro.

## 1. Introduction

Biofilms consist of either a single bacterium or a group of different bacteria that are present inside an extracellular polymeric substance. Biofilms formed by pathogens are a serious health concern as they are resistant to antibiotics, the immune defense systems of the host, and other external influences. The extracellular polymeric substance (EPS) plays a crucial role in the formation of biofilms and serves as a protective barrier against diverse environmental stressors, including antibiotics. This function of EPS confers a high level of resistance to pathogens, leading to the emergence of bacteria that are resistant to multiple drugs, extensively drug-resistant, and completely drug-resistant [1]. Biofilm formation affects human health in various ways, from infection of implants to wound infections. The Center for Disease Control (USA) estimates that 65% of infectious diseases in humans are due to biofilms formed by bacteria, while the National Institute of Health estimates it to be around 80%. The biofilms are resistant to many antimicrobial drugs, and the high rate of false negative results in bacterial cultures because of bacteria in the biofilm state further complicates antimicrobial therapy, leading to a severe impact on the affected individuals [2].

Biofilms are usually formed on the wounds in diabetic patients [3]. The wound-healing process normally consists of hemostasis, inflammation, proliferation, and remodeling and these stages overlap with each other [4]. In diabetes, the wound-healing process is impaired due to several factors. One the most important factor is biofilm formation, which acts as a mechanical barrier to antimicrobial agents and also interferes with the natural healing process [2]. Apart from this, increased blood glucose levels delay wound closure by interfering with cellular functions in the skin and peripheral neuropathy [5,6]. Additionally, peripheral vascular disease decreases wound healing due to altered blood flow and hypoxia [7,8]. There is also transepidermal water loss and less production of antimicrobial peptides due to the impaired differentiation of adipocytes from fibroblast [4].

Herbs and spices, alone or in combination, have been reported to have antibiofilm activities. Earlier studies suggest that clove, eucalyptus, and rosemary have varying effects against bacterial biofilms [9]. Interesting antibiofilm and anti-quorum sensing abilities have been reported in several desert plants, suggesting that these plants possess strong antimicrobial effects [10]. *Achillea fragrantissima* (yarrow) is commonly known as ‘Quysoom-aletri’ in Arabic and is widely found in Arab countries [11]. It is traditionally used as a medicinal tea for the treatment of various disorders, such as respiratory infections, digestive problems, eye infections, diabetes, and diarrhea [12]. This plant is rich in phenolic acids and a large number of flavonoids that are known to possess good antimicrobial effects.

This plant is widely used in Saudi Arabia as an antimicrobial and wound-healing agent (local source). It is believed to be highly effective in the healing of chronic wounds. Earlier reports show that the essential oil of this plant is active against several species of bacteria and also possesses antiviral properties [13,14,15]. Furthermore, several constituents present in *Achillea fragrantissima* are reported to have potent antimicrobial effects [16]. Hence, the current study was envisaged to assess the antibiofilm activity against MRSA and MDR-*P. aeruginosa* using a biofilm model induced through an excision wound in diabetic mice.

## 2. Results

### 2.1. LC-MS Analysis of the Achillea fragrantissima Methanolic Extract

**Figure 1:**
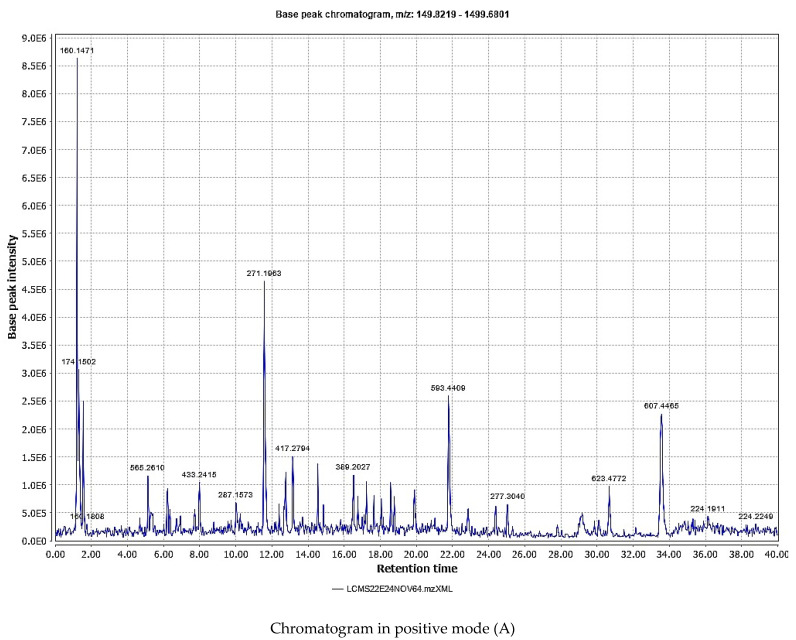

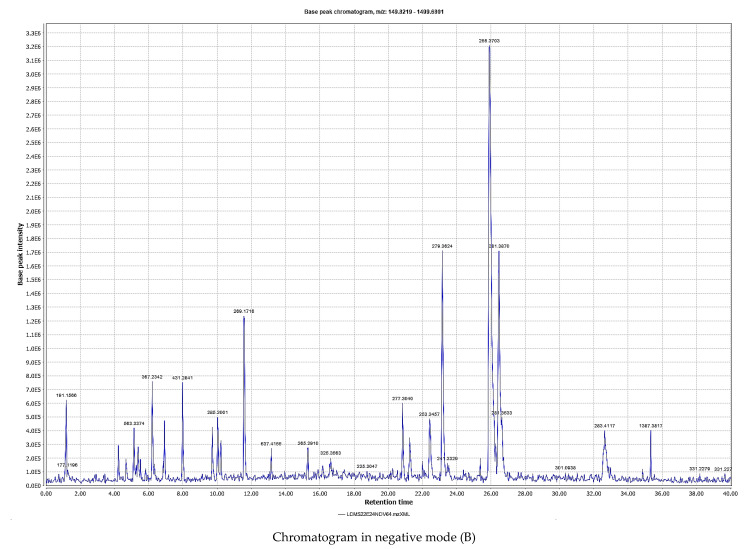
Chromatogram of the *Achillea fragrantissima* methanolic extract showing different peaks in positive mode (A) and negative mode (B) in LC-MS analysis.

A large number of molecules were identified in the extract through LC-MS analysis (Figure 1). In the positive mode, 24 molecules were suspected to be present in the extract, while in the negative mode, another 22 molecules were identified (Table 1 and Table 2).

### 2.2. Antibacterial Activity

The methanol extract of *A. fragrantissima* showed inhibitory effects against MRSA (MIC-256 µg/mL) and *P. aeruginosa* (MIC-512 mg/mL). The MBC concentration was 512 µg/mL for MRSA and 1024 mg/mL for *P. aeruginosa.* However, the effect was stronger against MRSA when compared to *P. aeruginosa*.

### 2.3. Antibiofilm Activity In Vitro

**Figure 2:**
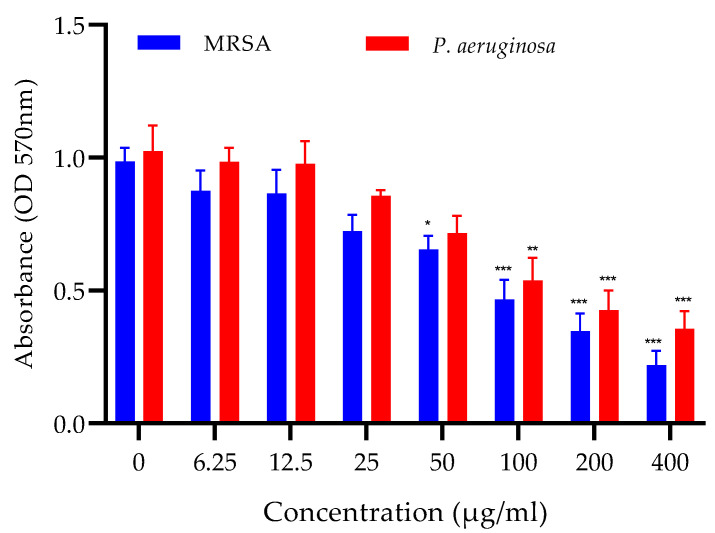
Antibiofilm activity of methanolic extract of *A. fragrantissima* in vitro against MRSA and *P. aeruginosa* in crystal violet assay. Absorbance is measured for the quantification of crystal violet. Amount of crystal violet is directly proportional to the biofilm formation. Bars represent mean ± SEM, n = 3., * *p* < 0.05, ** *p* < 0.01, *** *p* < 0.001 compared to 0 µg/mL concentration.

The methanolic extract of *Achillea fragrantissima* showed antibiofilm activity against both MRSA and *P. aeruginosa*. The antibiofilm activity was observed at a minimum concentration of 50 µg/mL against MRSA, while with *P. aeruginosa,* there was a relatively lesser effect and the antibiofilm effect was seen from 100 µg/mL onwards. The effect against both pathogens was concentration-dependent (Figure 2).

### 2.4. Skin Irritation Test

No visible irritation or inflammation was seen up to 72 h after the extract application in the form of ointment.

### 2.5. Physicochemical Properties of the Ointment

The extract ointment showed good stability. The diffusing ability, homogeneity, spreading ability, and washability were checked and found to be satisfactory.

### 2.6. Wound-Healing Activity against MRSA-Induced Biofilm Formation

Local application of the methanolic extract of *Achillea fragrantissima* ointment formulation enhanced the contraction of biofilm-formed wounds in mice compared to the control, indicating the increased healing of wounds. The higher concentration of the extract (100 mg/g) significantly increased wound contraction from the 8th day onwards (*p* < 0.05) when compared to the control, while no such effect was observed with the lower concentration (50 mg/g). The lower concentration showed a significant increase in wound contraction on the 16th and 20th day when compared to the control, suggesting a weaker effect as compared to the higher concentration. 

**Figure 3:**
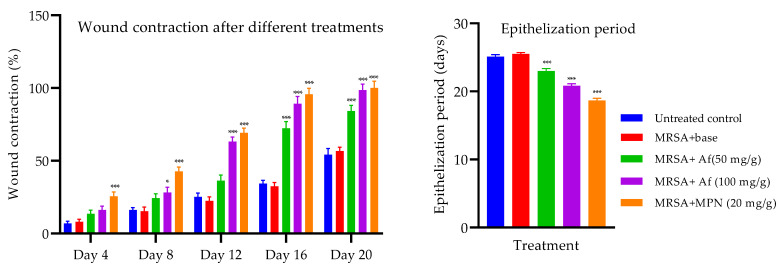
Wound contraction and epithelization period after different treatments on excision wound in MRSA-induced biofilm. Wound contraction indicates decrease in the wound area as compared to the initial wound area. Day of epithelization shows complete healing of wounds without any raw wound. Bars represent mean ± SEM, n = 6, * *p* < 0.05, *** *p* < 0.001 compared to the MRSA + base treatment. Af—*Achillea fragrantissima* formulation; MPN—mupirocin.

The mupirocin ointment was highly effective in healing the wounds from the 4th day onwards, and its effect was markedly stronger when compared to either concentration of methanolic extract of *Achillea fragrantissima* formulation. No significant difference was seen between the untreated group and base treatment groups, confirming the inert nature of the base used. The epithelization period was significantly reduced in all the treatment groups in comparison to base treatment (Figure 3).

### 2.7. Wound-Healing Activity against P. aeruginosa-Induced Biofilm Formation

**Figure 4:**
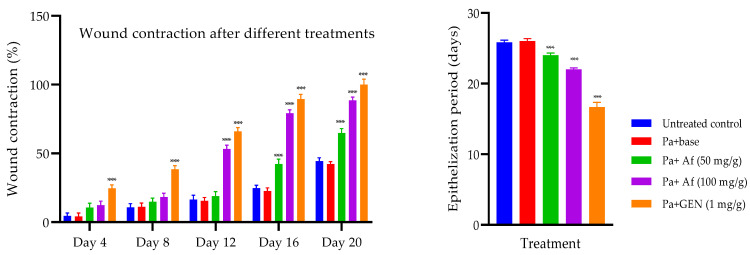
Wound contraction and the epithelization period after different treatments on excision wound in *P. aeruginosa*-induced biofilm. Wound contraction indicates a decrease in the wound area as compared to the initial wound area. Day of epithelization shows complete healing of wounds without any raw wound. Bars represent mean ± SEM, n = 6, *** *p* < 0.001 compared to the base treatment. Af—*Achillea fragrantissima* formulation; GEN—gentamicin.

The application of the prepared formulation of *A. fragrantissima* improved the contraction of biofilm-formed wounds in mice when compared to the control. The higher concentration of the extract (100 mg/g) significantly showed wound contraction from the 12th day onwards when compared to the control. A lesser effect was observed with the lower concentration of the extract formulation (50 mg/g). However, the lower concentration exhibited a significant improvement in wound contraction on the 16th and 20th day when compared to the control. This shows a weaker effect of the lower concentration (50 mg/g) as compared to the higher concentration (100 mg/g). The antibiotic gentamicin (1 mg/g) was highly effective in healing wounds from the 4th day onwards and had a superior effect when compared to the higher and lower concentrations of *A. fragrantissima* formulation. No significant difference was observed among the untreated group and base-treated groups showing the inert effect of the ointment base. The effect on the epithelization period in MDR-*P. aeruginosa* biofilm on the excision wound was similar to that observed after MRSA infection (Figure 4).

The bacterial load in the wounded tissue on 20th day after treatment with the extract formulation was significantly reduced when compared to the base-treated control. However, the *P. aeruginosa* count was not significant in the case of lower concentration (50 mg/g), whereas the higher concentration of the formulation significantly reduced the bacterial count. The antibiotic treatment with mupirocin (20 mg/g) and gentamicin (1 mg/g) had a significant effect in reducing bacterial load compared to the control (Table 3).

### 2.8. Histological Study

**Figure 5:**
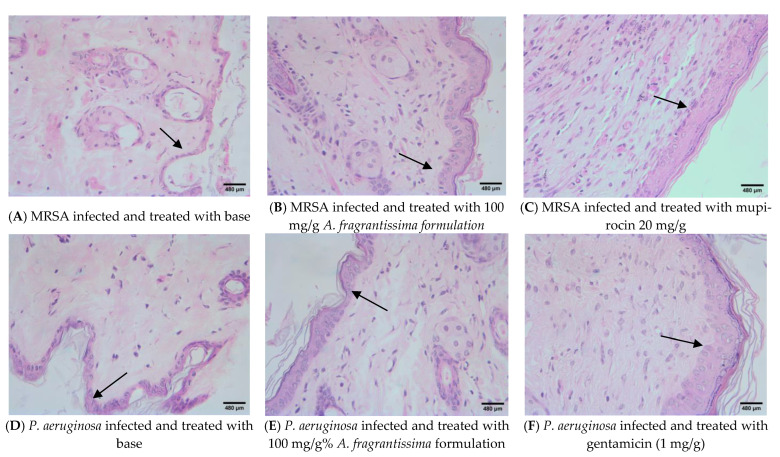
Histological changes (400×) in the wounded tissue after different treatments. Arrows indicate regenerated skin epithelium. The thickness of the epithelium is lowest in animals treated with base-treated controls (**A**,**D**). The thickness of regenerated epithelium was higher after extract treatment in both MRSA (**B**) and *P. aeruginosa* (**E**) infected wounds and the regenerated epithelial height was maximum in animals treated with respective antibiotics (**C**,**F**).

Macroscopic examination of the wound on the day of epithelization showed complete healing of the wound without any exudates (Figure S1). Histological microscopic examination showed increased regeneration of skin epithelium when compared to the control in both MRSA-infected and MDR-*P. aeruginosa*-infected wounds (Figure 5). In the base-treated control, infection with MRSA delayed the regeneration of skin epithelial tissues. Application of the *A. fragrantissima* extract treatment (100 mg/g) increased the thickness of epithelial tissues as compared to the base-treated group, though complete regeneration was not visible. The epidermal thickness was not measured quantitatively, and qualitative observation was used to indicate the difference. Treatment with mupirocin was highly effective in causing skin epithelial regeneration, and the tissues resembled a normal unwounded tissue. Infection with *P. aeruginosa* delayed the development of epithelial tissue, confirming the macroscopic observations. In comparison to the effect of *A. fragrantissima* extract treatment (100 mg/g) on MRSA-infected wounds, the effect on *P. aeruginosa*-infected wound seemed to be less as the thickness of regenerated epithelial was thinner. Gentamicin (1 mg/g) was highly effective in increasing skin thickness as compared to other treatments. After treatment with the extract for 20 days, the sections showed scanty presence of inflammatory cells because the wounds were almost healed in the treatment groups as compared to the control. Treatment with the extract increased the proliferation of fibroblasts, indicating enhanced collagen deposition in the tissue.

#### Cytotoxicity Study of Extract In Vitro

**Figure 6:**
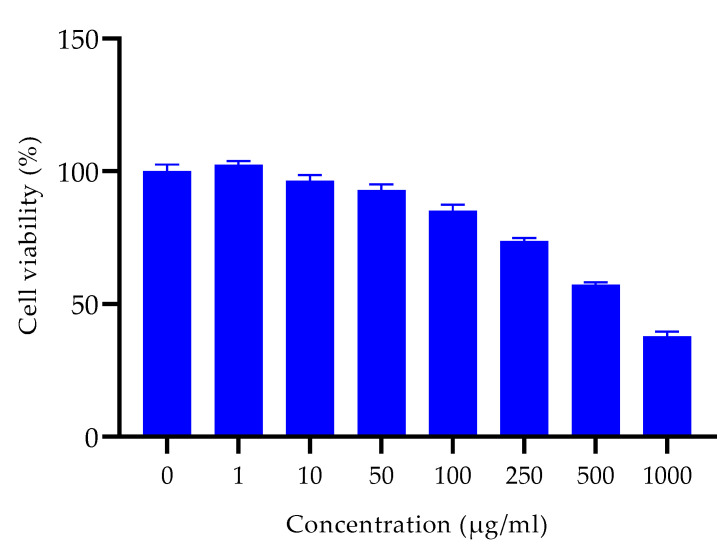
Cell viability of HaCaT cell lines treated with different concentrations of the extract.

The extract was safe and did not show profound toxicity on HaCaT cell lines in vitro. The IC_50_ value of the extract was 647.3 µg/mL (Figure 6).

## 3. Discussion

The current study was an attempt to explore the biological effects of desert plants. As mentioned earlier, desert plants are known to possess a wide range of effects, many of which have not yet been explored. The evaluation of antibiofilm activity was undertaken based on local belief in Saudi Arabia that this plant is an excellent antimicrobial agent. The results of the current study confirmed the antimicrobial effects and its antibiofilm activity, suggesting that this plant may be a potential antimicrobial agent from a natural source.

The study involved both in vitro and in vivo evaluations to confirm its beneficial antibiofilm effect. We selected a mouse model that has been reported earlier as a simple method for the study of the antibiofilm effect [17]. The method involved the use of diabetic mice with an application of preformed biofilm. Though the method used described the use of MRSA to induce the biofilm, it worked equally well with MDR-*P. aeruginosa*. The antibiofilm effect was determined using different parameters that included histological studies to confirm the healing of excised wounds containing biofilm. The regeneration of skin epithelial tissue after different treatments indicates increased wound healing. Infection and biofilm formation interferes with the normal healing process due to the formation of degenerative enzymes and toxins that causes chronic inflammation [18]. Different parameters, such as epithelial height, density of capillaries, and collagen deposition in the wound, are used to determine the wound-healing process. Of these, the most widely used parameter is epithelial regeneration [19]. In the current study, *Achillea fragrantissima* extract increased the regeneration of skin epithelium, suggesting its wound-healing action. However, the regenerated skin epithelium was less prominent as compared to that observed with the standard antibiotics mupirocin and gentamicin, which confirmed a relatively lesser antibiofilm effect of *Achillea fragrantissima* extract when compared to mupirocin and gentamicin. Since the antibiofilm effect and wound-healing effect of *Achillea fragrantissima* was related, the contribution of other mechanisms to wound healing, such as the antioxidant and proliferative effects of the extract, can be ruled out.

*Achillea fragrantissima* extract in the form of an ointment formulation showed good antimicrobial and antibiofilm effects, as indicated by the increased healing of excision wounds and a decrease in microbial load at the end of the experiment. The ointment base was selected based on earlier studies that suggested it is suitable for the preparation of formulations using methanolic extracts [20]. The formulation had a better effect against MRSA in comparison to *P. aeruginosa,* and these results were expected as plant products are usually more active against Gram-positive bacteria as compared to Gram-negative bacteria. The Gram-negative bacteria are known to be resistant to antimicrobial agents due to their cell wall structure which contains a lipopolysaccharide layer and periplasmic space [21]. However, its effect against both of these pathogens suggests its potential as a broad-spectrum antibacterial agent. The ointment formulation did not induce any irritation or inflammation on mouse skin.

*Achillea fragrantissima* contains a large number of chemical constituents. An LC-MS analysis revealed the presence of 47 constituents, and many of these have been reported for antimicrobial activity against many different pathogens. Among these, nicotine is reported to possess antibacterial and antifungal activities [5]. Vitexin, a polyphenolic compound in the plant, is reported for antibiofilm activity against *P. aeruginosa* [22]. Fornesol, which is a sesquiterpene alcohol present in *Achillea fragrantissima,* has also been reported for antibacterial and antifungal effects [23]. Chalcone, naturally present in the plant, and many of its derivatives have been reported to possess excellent antibacterial activity against a variety of bacteria, including *P. aeruginosa* [24]. Plants containing fortunelin, which is also found in *Achillea fragrantissima,* are reported for antibacterial and antiviral effects [25]. Similarly, quinic acid has been previously reported for excellent antibacterial effects [26]. *Achillea fragrantissima* contains catechins such as epicatechin, and catechins are known antibacterial compounds [27]. Acacetin, a simple flavone [28], formononetin [29], pentachlorophenol [30], and linolenic acid [31] are the other constituents reported for antimicrobial effects. It should also be noted that *Achillea fragrantissima* and many of the identified constituents present in it have been reported for antioxidant activities. It is well known that antioxidants increase the healing of wounds through free radical scavenging effects [32,33]. As mentioned above, the antibiofilm effect of *Achillea fragrantissima* was closely related to the wound-healing effect, and the results suggest that there may be a negligible contribution of antioxidant action to the wound-healing effect.

The following are the limitations of the study that can be addressed with further investigations. The present study identified different chemicals present in the extract. However, a detailed study with isolated compounds from the extract is required that may lead to the identification of the exact number(s) of chemical constituents that are involved in the antibiofilm and the wound-healing actions of the plant. The results of the current study rule out the contribution of other mechanisms such as antioxidant, anti-inflammatory, and proliferative effects of the plant extract because the wound-healing and antibiofilm effects were related. However, no study was performed to determine these effects of the prepared extract. The histological examination of the tissue was performed to support the macroscopic, and antimicrobial findings. Histology was not studied in detail with respect to the effect on different processes involved in wound healing. Further studies on the effect of plant extract on proliferative genes, such as the vascular endothelial growth factor (VEGF) and transforming growth factor β-1 (TGF-β-1), and detailed histological examination using different stains, such as Masson’s trichrome stain to study collagen deposition, may provide information about the proliferative effects of the extract [34]. Further studies on the contribution of antioxidant effects through increased levels of antioxidant enzymes such as catalase and superoxide dismutase should also be carried out for the determination of free radical scavenging activity of the plant extract [35].

## 4. Materials and Methods

### 4.1. Chemicals and Micro-Organisms

The chemicals were purchased from local suppliers. The chemicals were of analytical grade. The pathogens MRSA (ATCC43300) and MDR-*P. aeruginosa* (ATCC 27853), cultured in the laboratory, were employed. 

### 4.2. Animals

Adult albino Swiss mice weighing between 25 and 28 g and maintained under standard conditions were used. The experimental protocol was approved by the Ethical Research Committee of Shaqra University (No. ERC SU_20220066). All procedures used were in accordance with the ARRIVE guidelines [36].

### 4.3. Plant Extraction and LC-MS Analysis

The whole plant was collected in October 2022 and was identified by a botanist in the college. A voucher specimen (No. SU/CAMS/08/2022), the plant was dried under the shade and extracted using 90% *v*/*v* methanol in a Soxhlet extractor [37]. The extract was dried using a rotavapor and the yield was 16.24% *w*/*w*. 

The LC-MS analysis was conducted using a Waters (Milford, MA, USA) LC instrument (XEVO-TQD#QCA1232) with a C_18_ column (SUNFIRE C_18_, 250 mm × 2.1 mm, 2.6 µm) with a flow rate of 0.2 mL/min and 280 nm detection. The solvent system consists of solvent A (acetonitrile) and solvent B (ammonium formate buffer). The HPLC conditions and gradient table used are given (Table 4 and Table 5). Spectra were recorded in negative and positive ionization modes between m/z 150 and 2000.

### 4.4. Antibacterial Activity

The extract was evaluated to determine its minimum inhibitory concentration (MIC) and minimum bactericidal concentration (MBC) using standard methods described elsewhere [38].

### 4.5. In Vitro Antibiofilm Activity

Bacterial cultures of MRSA or MDR-*P. aeruginosa* (10^6^ CFU/mL) in Luria Bertani (LB) broth grown in microtiter were used. The antibiofilm activity was determined with the crystal violet assay [39]. Different concentrations of plant extracts were added to the wells, ranging from 6.25 µg/mL to 400 µg/mL. The microtiter plates were incubated at 37 °C for 24 h. The planktonic cells were discarded, and the wells were rinsed three times with distilled water. Crystal violet (20 μL) was added to the wells for staining, followed by rinsing with potassium phosphate buffer (10 mM) and drying. Crystal violet was solubilized using 96% *v*/*v* ethanol, and the absorbance was measured at 570 nm.

### 4.6. Preparation of the Extract Ointment

The *Achillea fragrantissima* extract was formulated into an ointment of 2 different concentrations (50 mg/g of the base and 100 mg/g of the base). A fusion method using a base made from liquid paraffin, emulsifying wax, and soft paraffin was employed to prepare the extract ointment [20]. The physicochemical properties such as spreadability, washability, diffusion, and stability were evaluated [40].

### 4.7. Skin Irritation Test in Mice

The ointment formulation was applied to the depilated area on the mouse skin. The ointment was allowed to remain in place for 72 h and signs of irritation or inflammation, such as redness and edema, were recorded.

### 4.8. Induction of Diabetes in Mice

Diabetes (type II) was induced using streptozocin and nicotinamide [41]. Animals fasted for 12 h and were injected intraperitoneally with nicotinamide (240 mg/kg), followed 15 min later by intraperitoneal injection of streptozocin (100 mg/kg). The nicotinamide and streptozocin were prepared using normal saline as a vehicle [42]. The volume of the injection was adjusted to 1 mL/100 g. After 72 h of streptozocin injection, animals were fasted for 12 h, followed by the determination of their blood glucose levels. Mice with a blood sugar level of 150 mg/dL or more were selected for the determination of antibiofilm activity. The animals were allowed free access to water and feed throughout the experimental period. Precautions were exercised to avoid transmission of infection, and animals were monitored carefully for mortality.

### 4.9. Antibiofilm Activity In Vivo

For the determination of the antibiofilm activity of the formulation, a method described earlier was used [17]. The pathogens were grown on a coverslip immersed in LB broth in a culture bottle inoculated with either MRSA or *P. aeruginosa*. The biofilm formation on the coverslip was established through coverslip assay using crystal violet [43]. 

The excision wounds were induced by depilating skin at the back of the animals under anesthesia [44]. A coverslip containing the biofilm was placed over the wounded surface along with 100 µL of broth culture (10^6^ CFU/mL) of either MRSA or MDR-*P. aeruginosa.* After 72 h, the biofilm formation on the wound was confirmed in at least 10% of the initial number of animals by excising the thin layer on the wound, followed by modified Gram staining. The animals were then grouped into two major groups, one for MRSA and the other for MDR-*P. aeruginosa.* Each of these groups had five subgroups containing twelve animals at the end of the experiment. These five groups were treated as follows: group I was untreated, group II was treated with the ointment base, group III and group IV received an application of the extract ointment at 50 mg/g and 100 mg/g, respectively, while the fifth group received the standard antibiotic (mupirocin 20 mg/g or gentamicin 1 mg/g). The wound area was measured at 4-day intervals for 20 days in 6 animals. 

The wound contraction (%) was calculated using the formula [45]

Wound contraction (%) = (Total wound area − present wound area/Total wound area) × 100

The total wound area is the area on Day 0 (1 cm^2^) and the present wound area refers to the day on which the wound area was measured (4th, 8th, 12th, 16th, and 20th day). The wound area was measured by tracing the wound using a transparent sheet and superimposing it on a graph sheet [46].

At the end of the 20th day, these animals were sacrificed, the bacterial count (CFU/g) was determined, and the tissues were also subjected to histological studies using H and E stain [47]. The thickness of the regenerated skin epithelium was qualitatively observed and the thickness of the epithelium in the base-treated animals were compared with the treated animals. The sections were observed for the presence of inflammatory cells and fibroblasts. 

The remaining six animals were given treatment until the wound scar disappeared to indicate the day of complete epithelization. The epithelization period refers to the day on which there was a falling of the scar, leaving no raw wound [48]. The re-epithelization was indicated by absence of any exudates, and growth of hair on the edges of the wound. Histological examinations on the wound on the day of epithelization were not carried out due to ethical issues involved in sacrificing a large number of animals. Furthermore, the regenerated epithelium would appear the same in the all the treatment groups as the tissue has to be taken from the healed wounds.

### 4.10. Cytotoxic Assay In Vitro on HaCaT Cell Lines

Cytotoxicity of the extract on the HaCaT cell line was evaluated with the SRB Assay. The cells cultured in a DMEM medium supplemented with 10% FBS and 1% antibiotic solution were used. The extract was tested at different concentrations (1–1000 µg/mL). After incubation for 24 h, 100 µL of tricolor acetic acid (10% *w*/*v*) was added to each well and incubated for 1 h, followed by washing with distilled water and air drying. Sulforhodamine (SRB) solution (0.04% *w*/*v*) was added to each well and left for 1 h. After incubation, the plate was washed with acetic acid (1% *v*/*v*) to remove the unbound dye and air-dried. Tris base solution (pH = 10. 5) was added to the wells and shaken for 10 min on an orbital shaker to solubilize the protein-bound dye and read at 510 nm.

### 4.11. Statistical Analysis

The results are shown as mean ± SEM. The number of recordings and level of significance is mentioned in the footnotes. Analysis was performed using SPSS software (version 20 for Windows). One-way analysis of variance (ANOVA) with Tukey’s post-test was used to determine statistical differences between the different treatments. A probability value of 0.05 or less was indicative of a statistically significant difference.

## 5. Conclusions

The *Achillea fragrantissima* methanolic extract showed antibacterial and antibiofilm effects against both MRSA and MDR-*P. aeruginosa*. The effect was relatively lesser against MDR-*P. aeruginosa* as compared to MRSA. The LC-MS analysis of the extract showed the presence of 47 different phytoconstituents, and many of these are known to have antimicrobial activities. A detailed investigation of the effect of these phytoconstituents is required to determine their contribution to the antibiofilm and wound-healing effects of the plant extract. Furthermore, study of the effect of extract on polymicrobial biofilms on wounds may provide more insight into the activity of the plant extract.

## Figures and Tables

**Table 1:** List of suspected molecules in positive mode.

Score	Compound Name	Ion	Formula	Exact Mass	Observed Mass	Mass Diff
0.975	N-alpha-Acetyl-L-ornithine	positive	C_7_H_14_N_2_O_3_	174.1	174.1502	−0.05
0.939	L(+)-Arginine	positive	C_6_H_14_N_4_O_2_	174.111	174.1502	−0.04
0.507	Canthaxanthin	positive	C_40_H_52_O_2_	564.396	565.2610	−0.87
0.782	(-)-Nicotine	positive	C_10_H_14_N_2_	162.115	391.2274	−229.11
0.677	L-beta-homotyrosine-HCl	positive	C_10_H_13_NO_3_	195.089	197.0278	−1.94
0.676	Vitexin	positive	C_21_H_20_O_10_	432.105	433.2415	−1.14
0.966	Pelargonidin chloride	positive	C_15_H_11_O_5_	271.06	271.1963	−0.14
0.203	3-Hydroxy-DL-kynurenine	[M + H]+	C_10_H_12_N_2_O_4_	224.21	224.2586	−0.05
0.927	N-Acetyl-Phytosphingosine	positive	C_20_H_41_NO_4_	359.303	361.1600	−1.86
0.908	1-Isothiocyanato-8-(methylsulfinyl)-octane	positive	C_10_H_19_NOS_2_	233.09	233.2006	−0.11
0.757	N1-Acetylspermine Trihydrochloride	positive	C_12_H_28_N_4_O	244.226	246.2257	−2
0.805	1-O-b-D-glucopyranosyl sinapate	positive	C_17_H_22_O_10_	386.121	389.2027	−3.08
0.8	Caffeine, Anhydrous	[M + H]+	C_8_H_10_N_4_O_2_	194.19	194.1934	0
0.935	Farnesol (mixture of isomers)	positive	C_15_H_26_O	222.198	222.2340	−0.04
0.926	Chalcone	positive	C_15_H_12_O	208.088	209.1754	−1.09
0.903	1-Isothiocyanato-8-(methylsulfinyl)-octane	positive	C_10_H_19_NOS_2_	233.09	234.2467	−1.16
0.96	Methyl Jasmonate	positive	C_13_H_20_O_3_	224.141	224.2249	−0.08
0.713	Fortunellin	positive	C_28_H_32_O_14_	592.179	593.4409	−1.26
0.69	Adenosine-5′-diphospho-glucose disodium salt	[M + H]+	C_16_H_25_N_5_O_15_P_2_	589.32	593.4409	−4.12
0.723	L-saccharopine	positive	C_11_H_20_N_2_O_6_	276.132	277.3040	−1.17
0.607	2′-Deoxycytidine-5′-diphosphate sodium salt	[M + H]+	C_9_H_15_N_3_O_10_P_2_	387.18	391.4299	−4.25
0.838	isorhamnetin-3-O-rutinoside	positive	C_28_H_32_O_16_	624.169	623.4772	0.69
0.86	Guanosine-5′-diphosphate-D-mannose sodium salt	[M + H]+	C_16_H_25_N_5_O_16_P_2_	605.34	607.4465	−2.11
0.88	3-Hydroxy-DL-kynurenine	[M + H]+	C_10_H_12_N_2_O_4_	224.21	224.2249	−0.01

**Table 2:** List of suspected molecules in negative mode.

Score	Compound Name	Ion	Formula	Exact Mass	Observed Mass	Mass Diff
0.856	D-(-)-Quinic acid	negative	C_7_H_12_O_6_	192.063	191.1566	0.91
0.901	(+)-Epicatechin	negative	C_15_H_14_O_6_	290.079	279.2157	10.86
0.618	Piperacillin sodium salt	negative	C_23_H_27_N_5_O_7_S	517.163	515.3142	1.85
0.754	UDP-glucose Disodium Salt	negative	C_15_H_24_N_2_O_17_P_2_	566.055	563.3374	2.72
0.869	Homoorientin	negative	C_21_H_20_O_11_	448.1	447.2126	0.89
0.938	Homoorientin	negative	C_21_H_20_O_11_	448.1	447.2800	0.82
0.946	Kaempferol-3-O-alpha-L-rhamnoside	negative	C_21_H_20_O_10_	432.105	431.2841	0.82
0.413	UDP-xylose	negative	C_14_H_22_N_2_O_16_P_2_	536.044	529.4208	−97.32
0.885	Acacetin	negative	C_16_H_12_O_5_	284.068	285.2001	−1.13
0.431	UDP-xylose	negative	C_14_H_22_N_2_O_16_P_2_	536.044	529.2183	6.83
0.935	Formononetin	negative	C_16_H_12_O_4_	268.073	269.1716	−1.1
0.422	Puerarin	negative	C_21_H_20_O_9_	416.11	415.3559	0.75
0.952	Pentachlorophenol	[M−H]−	C_6_HC_l5_O	266.34	265.2910	1.05
0.899	6-Phosphogluconic acid Barium salt hydrate	negative	C_6_H_13_O_10_P	276.024	277.3040	−1.28
0.895	2′-Deoxycytidine	negative	C_9_H_13_N_3_O_4_	227.09	227.2955	−0.21
0.93	2′-Deoxyinosine	negative	C_10_H_12_N_4_O_4_	252.085	253.3457	−1.26
0.982	gamma-Linolenic acid	[M−H]−	C_18_H_30_O_2_	278.43	279.3624	−0.93
0.976	2′-Deoxyinosine	negative	C_10_H_12_N_4_O_4_	252.085	255.3703	−3.29
0.973	Xanthosine	negative	C_10_H_12_N_4_O_6_	284.075	281.3870	2.69
0.916	gamma-Linolenic acid	[M−H]−	C_18_H_30_O_2_	278.43	281.3533	−4.98
0.944	Acacetin	negative	C_16_H_12_O_5_	284.068	283.4117	0.66
0.115	Kaempferide	negative	C_16_H_12_O_6_	300.063	301.0263	−0.96

**Table 3:** Bacterial count in the wounded tissue after the treatment.

Group	Log_10_ CFU/g of Tissue	
	MRSA	*P. aeruginosa*
Untreated control	5.20 ± 0.05	5.32 ± 0.035
Control (base)	5.38 ± 0.06	5.90 ± 0.076
*A. fragrantissima* ointment (50 mg/g)	4.07 ± 0.08 ***	4.36 ± 0.150
*A. fragrantissima* ointment (−100 mg/g)	2.84 ± 0.06 ***	3.27 ± 0.022 ***
^#^ Antibiotic	1.30 ± 0.04 ***	1.477 ± 0.059 ***

^#^ Antibiotic mupirocin (20 mg/g) for the MRSA-infected group, and gentamicin (1 mg/g) for *P. aeruginosa*-infected group. Data are mean ± SEM, n = 6, *** *p* < 0.001 in comparison to the control (base).

**Table 4:** HPLC conditions.

A%	0.0 H_2_O
B%	5.0 ACN
C%	0.0 MeOH
D%	95.0 0.1% Formic Acid in water
Flow (mL/min)	1.500
Stop Time (min)	5.0
Column Temperature (°C)	30.0
Min Pressure (Bar)	0.0
Max Pressure (Bar)	300.0

**Table 5:** The gradient table.

Time	A%	B%	C%	D%	Flow
0.00	0.0	5.0	0.0	95.0	1.500
1.00	0.0	5.0	0.0	95.0	1.500
6.00	0.0	30.0	0.0	70.0	1.500
12.00	0.0	60.0	0.0	40.0	1.500
16.00	0.0	60.0	0.0	40.0	1.500
20.00	0.0	80.0	0.0	20.0	1.500
26.00	0.0	5.0	0.0	95.0	1.500
30.00	0.0	5.0	0.0	95.0	1.500

## Data Availability

The supporting data will be available on request by writing to the corresponding authors (masad@su.edu.sa).

## Supplementary Materials

The following supporting information can be downloaded at: https://www.mdpi.com/article/10.3390/ijms24119774/s1.
