# Supplementary material for: Effect of Achillea fragrantissima Extract on Excision Wound Biofilms of MRSA and Pseudomonas aeruginosa in Diabetic Mice
Source: Int J Mol Sci. 2023 Jun 5;24(11):9774. doi: 10.3390/ijms24119774 (PMC10253793; doi:10.3390/ijms24119774)
Supplement: Supplementary file 1 [file ijms-24-09774-s001.zip › ijms-2422818-supplementary.pdf]

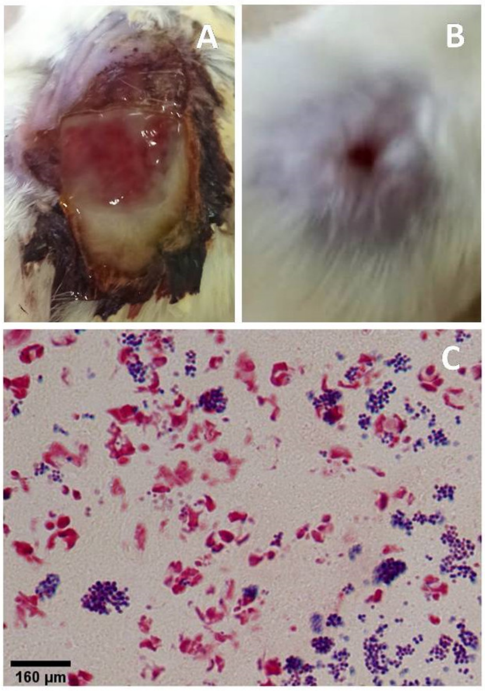

Figure S1. Representative images showing inflicted wound (**A**) and healed wound (**B**) on mouse skin. Histological examination (modified gram stain, 1000×) of wounded tissue indicating formation of biofilm on the wounded tissue (**C**).
